# The Role of ICG Angiography in Decision Making About Skin-Sparing in Pediatric Acute Trauma

**DOI:** 10.3389/fped.2022.851270

**Published:** 2022-03-15

**Authors:** Tao Han, Buhao Sun, Weidong Wang, Jie Cui, Weimin Shen

**Affiliations:** Department of Burns and Plastic Surgery, Children's Hospital of Nanjing Medical University, Nanjing, China

**Keywords:** indocyanine green angiography, trauma, necrosis, perfusion, children

## Abstract

**Background:**

Indocyanine green (ICG) angiography has proven useful in assessing skin flap perfusion in plastic and reconstructive surgeries. This research aimed to explore its role in decision making about skin-sparing in children's acute trauma.

**Methods:**

A total of 19 patients suffering with acute trauma from January 2019 to September 2021 were retrospectively assessed. Both ICG angiography and clinical judgment were performed to evaluate skin tissue viability. The intraoperative decisions for each case depended on the specific condition of the traumatic wound, including tissue perfusion, skin defect area, and location of the wound. Postoperative vascular imaging software was used to quantify the tissue perfusion, and the duration of postoperative follow-up was from 6 to 18 months.

**Results:**

Among them, 18 (94.7%) patients experienced treatments according to ICG angiography and did not develop postoperative necrosis. One case with right forearm trauma suffered from partial necrosis. Hypertrophic scar and local infection were the independent complications, which were managed by symptomatic treatment.

**Conclusion:**

ICG angiography may reduce the risk of postoperative necrosis and renders a promising adjunctive technique for surgeons to make reasonable decisions in skin sparing in acute pediatric trauma.

## Introduction

The evaluation of skin tissue viability is the key to the management of children's acute trauma and is generally associated with the prognosis of local appearance or function (1, 2). An appropriate debridement of skin tissues with hypoperfusion, which can remarkably reduce the occurrence of postoperative necrosis, however, is mainly based on subjective experience. Therefore, decision making about skin sparing is a test for young surgeons, especially in cases of laceration or avulsion injury, that create a state of vasoconstriction intraoperatively (3, 4). Due to inaccurate judgment and individual differences, local necrosis often occurs after operation, resulting in surgical complications, such as scar hyperplasia, delayed wound healing, and infection.

Intraoperative indocyanine green (ICG) angiography has been widely used in assessing skin flap perfusion in various kinds of plastic and reconstructive surgeries (5–8). It can be an efficient adjuvant to enhance the surgeon's judgment of skin tissue viability and significantly decrease the odds of local necrosis after flap surgery. Moreover, an increasing amount of research focuses on the application of ICG angiography in both burn depth estimation and precise marking for burn excision (9–11). However, the current clinical study of ICG angiography on acute trauma surgery is extremely limited. Therefore, this study aimed to characterize the efficacy and safety of ICG angiography in decision making about skin sparing in children's acute trauma.

## Patients and Methods

We retrospectively assessed the clinical features, management, and follow-up of 19 patients suffering with acute trauma. All of the included cases were treated in our center between January 2019 and September 2021. Skin tissue viability was evaluated by both ICG angiography and clinical judgment (capillary refill, skin color, and bleeding of wound margin). Inclusion criteria included (1) hypoperfusion by clinical judgment, (2) the traumatic wound involving only skin and soft tissue, and (3) more than 6 months of follow-up. Exclusion criteria included (1) patients having undergone previous intervention and (2) a history of iodine allergy.

Under general anesthesia, 2.0–4.0 ml ICG (concentration: 2.5 mg/ml, Dandong Yichuang, China) was applied intravenously in 10 s followed by a 10-mL saline bolus injection. The maximum dose of ICG was 0.5 mg/kg per session. It took 20–30 s to observe the full effect of fluorescence, and then the area of skin tissue perfusion was visualized by using a fluorescence imaging system (Mingde Medical Diagnosis Inc., Langfang, China). Subsequently, real-time ICG angiography was continuously conducted for 5–10 min. The percentage of wound skin viability was estimated by viable skin area/total trauma area (cm^2^). The interval should be more than 20 min if repeated ICG angiography is required. The intraoperative decisions for each case depended on the specific condition of the traumatic wound, including tissue perfusion, skin defect area, and location of the wound. Tissue with hypoperfusion was excised when identified by ICG results.

**Figure 1:**
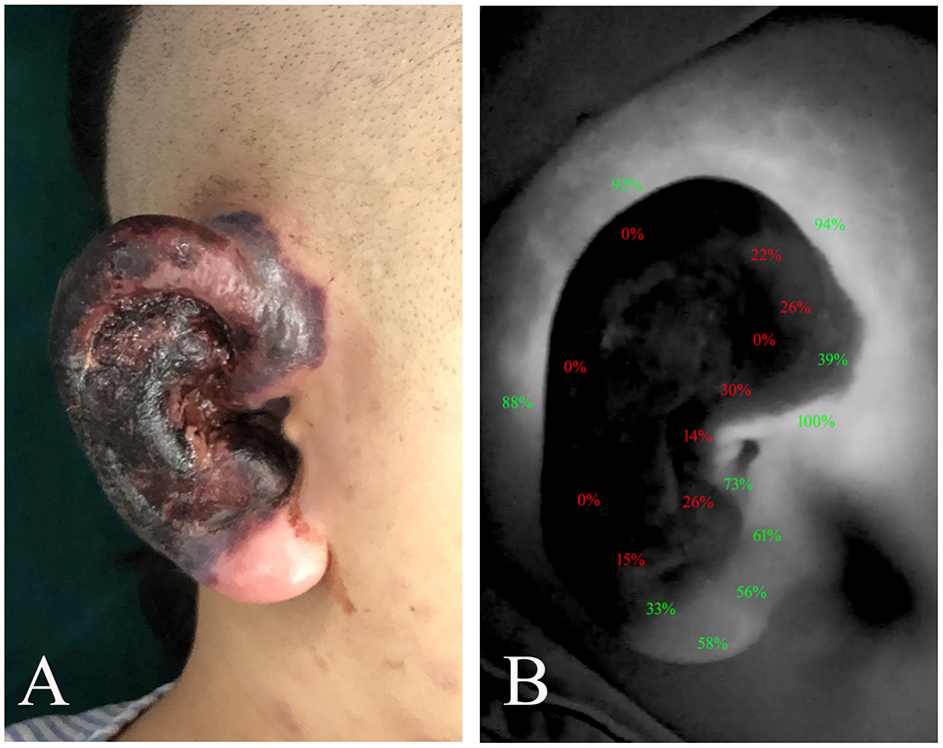
The intraoperative perfusion area was assessed by ICG angiography, and postoperative vascular imaging software was used to quantify the tissue perfusion at random points. **(A)** Clinical appearance of right ear with crush injury. **(B)** The percentage in the image represented tissue perfusion compared with the maximal area of perfusion, which was set as 100%. A perfusion intensity of <33% of maximal perfusion in the trauma site was regarded as an indicator of hypoperfusion. ICG, indocyanine green.

Moreover, postoperative vascular imaging software (Version 1.0, Mingde Medical Diagnosis Inc., Langfang, China) was used to quantify the tissue perfusion at a random point (Figure 1). A perfusion intensity of <33% of maximal perfusion in the trauma site was regarded as an indicator of hypoperfusion (7, 12). For all cases enrolled in this study, the duration of postoperative follow-up was from 6 to 18 months.

## Results

**Figure 2:**
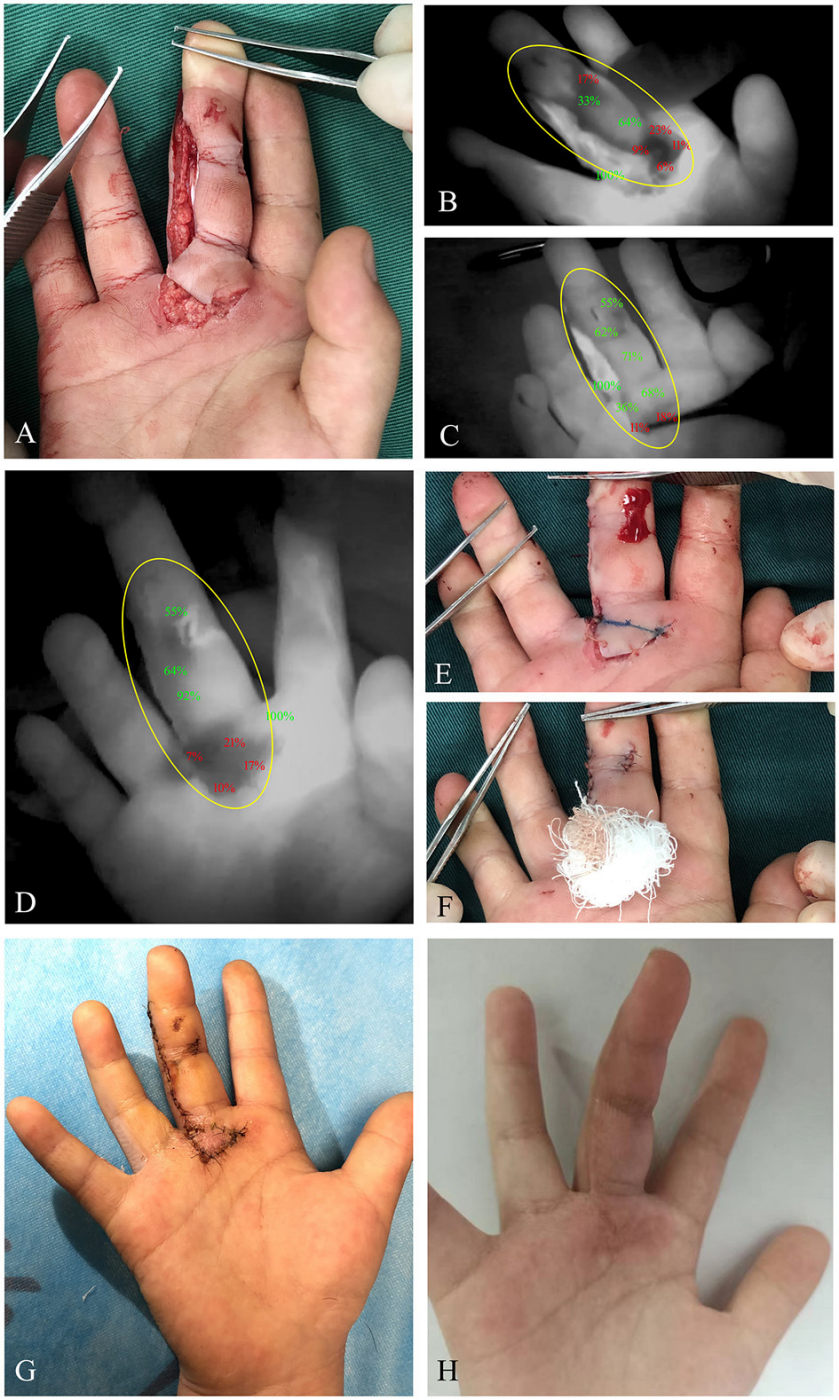
A 6-year-old female patient suffered with a cut of right middle finger caused by an electric fan. **(A)** Clinical appearance of the right middle finger with retrograde skin avulsion injury. **(B)** Immediate ICG angiography indicated diminished ICG uptake of proximal wound portion. **(C)** After 5-min observation, hypoperfusion of the proximal wound edge was still detected. **(D)** With the wound closure, ICG angiography was performed again to evaluate the skin perfusion, demonstrating persistent hypoperfusion of the proximal wound portion. **(E)** The proximal wound portion with poor capillary refill was marked under angiography. **(F)** With resection of the poorly perfused tissue, a reverse full-thickness skin graft was performed in this area. **(G)** Fourteen days postoperatively, ideal skin survival was obtained. **(H)** Clinical appearance at the third month postoperatively. *Yellow circle*, region of trauma. ICG, indocyanine green.

**Figure 3:**
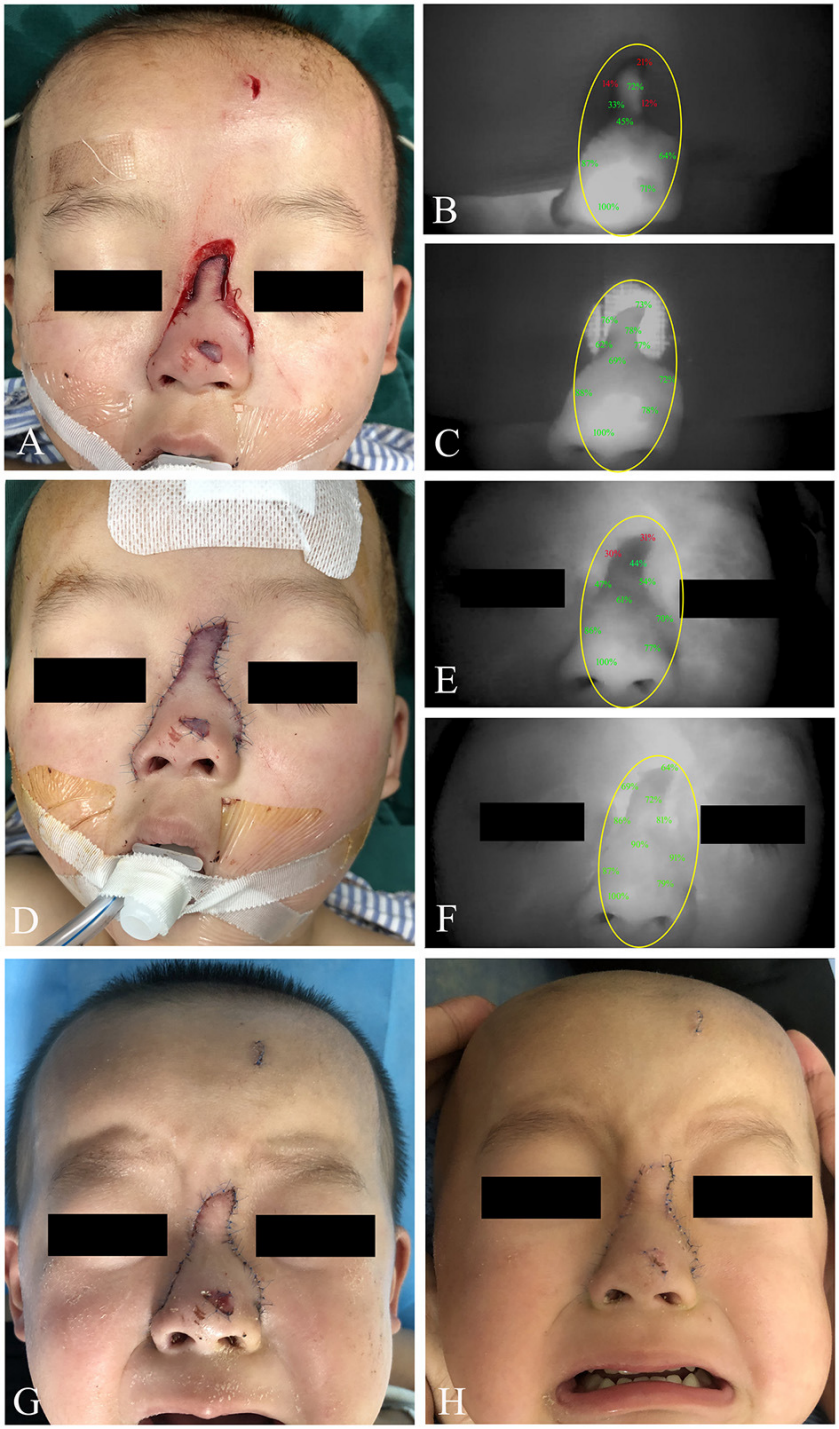
A 2-year-old male patient suffered with nose trauma caused by running against a glass door. **(A)** Clinical appearance of the distal wound edge with unhealthy color and poor capillary refill. **(B)** Immediate ICG angiography indicated diminished ICG uptake of the distal wound edge, which was consistent with clinical finding. **(C)** With 5-min continuous observation, improved perfusion of the distal wound was demonstrated by ICG angiography. **(D)** According to ICG findings, suture *in situ* with skin sparing was chosen to perform for the wound. **(E)** The wound was sutured in place, and ICG angiography was repeated immediately, demonstrating hypoperfusion again of the distal wound portion. **(F)** An acceptable perfusion was achieved after 5-min observation. **(G)** Four days postoperatively, clinical appearance with uneventful healing was obtained. **(H)** Clinical appearance at the 10th day postoperatively. *Yellow circle*, region of trauma. ICG, indocyanine green.

In this study, the male:female ratio was 13:6 with an age range of 2–10 years. The vast majority (14 cases, 73.7%) suffered with craniofacial trauma. Time to intraoperative ICG perfusion was from 15 to 75 s. A summary of clinical features of this study population is shown in Table 1. Precise marking for tissue with hypoperfusion was shown by excision ICG angiography. In 12 of 19 cases (63.2%), the skin viable portion judged by clinical finding was larger than that detected by ICG angiography (Figure 2). ICG angiography indicated acceptable perfusion in three cases (15.8%), which were determined to be an at-risk area by clinical judgment (Figure 3). Besides this, clinical findings were in accordance with ICG angiography in four cases (21.1%). Notably, 18 (94.7%) patients experienced appropriate treatment according to ICG angiography and did not develop postoperative necrosis. The case with partial necrosis was a fat boy. Part of the wound tissue with pale color and poor capillary refill was observed from clinical manifestation, and we performed resection of the tissue with hypoperfusion according to ICG assessments. A local skin flap with a thick fat layer was used to cover the defect of the wound, and postoperative skin necrosis was shown in the distal edge of the flap.

**Table 1:** Clinical features of 19 cases with acute trauma.

**Caes No**.	**Gender**	**Age (yr)**	**Location of trauma**	**Skin viable portion (%)**		**Treatment**	**Postoperative necrosis**	**Follow-up (month)**
				**Clinical judgment**	**ICG angiography**			
1	M	2	Nose	85	100	Suture *in situ* with skin-sparing	None	12
2	F	5	Left face	100	100	Suture *in situ* with skin-sparing	None	6
3	F	6	Left calf	90	82	Suture *in situ* after partial resection	None	12
4	M	3	Scalp	100	91	Suture *in situ* after partial resection	None	18
5	M	4	Left forehead	94	82	Local flap after partial resection	None	10
6	M	4	Bilateral upper eyelid	100	100	Suture *in situ* with skin-sparing	None	18
7	M	7	Lower lip	100	92	Local flap after partial resection	None	10
8	F	6	Right middle finger	85	80	Skin graft after partial resection	None	14
9	F	7	Upper lip	95	90	Suture *in situ* after partial resection	None	12
10	M	10	Left eyebrow	100	100	Suture *in situ* with skin-sparing	None	6
11	M	8	Right forearm	93	85	Local flap after partial resection	Partial necrosis	12
12	F	9	Right ear	35	20	Subtotal resection	None	12
13	M	3	Right ring finger	82	100	Skin graft after partial resection	None	18
14	M	6	Nose	100	95	Local flap after partial resection	None	18
15	M	7	Left upper arm	85	100	Suture *in situ* after partial resection	None	12
16	M	8	Neck	100	100	Suture *in situ* with skin-sparing	None	10
17	M	9	Left index finger	100	91	Distal-based thenar flap after partial resection	None	14
18	F	5	Right face	96	90	Local flap after partial resection	None	10
19	M	3	Right forehead	95	87	Suture *in situ* after partial resection	None	8

*ICG, indocyanine green; M, male; F, female*.

All cases underwent this investigation without any reports of allergic reactions, nausea, hematoma, or ICG-related pigmentation. Some minor complications were observed in this cohort, including two cases of hypertrophic scar and one case of local infection, which were managed by symptomatic treatment.

## Discussion

Characteristics owned uniquely by children make management of acute trauma more challenging. Distinguished from adults, children have less soft tissue to absorb the transmission of traumatic energy, leading to deeper injury and a larger extent of skin involvement (13, 14). Inaccurate acquisition of history and incompliant physical examination interfere with the surgeon's estimation of injury severity. Moreover, severe pain and crying may induce vasospasm and poor perfusion in the wound. For young surgeons, one of the major problems is how to determine the accurate extent and perfusion of involved skin tissue. As a result, optimizing the decision on skin sparing in acute trauma surgery to minimize any negative impact on postoperative appearance is becoming increasingly important.

Some non-invasive techniques are reported in wound management for the estimation of depth and severity, including infrared thermography, laser doppler imaging, and ICG angiography. Infrared thermography is easily operated and permits accurate evaluation of wound viability much earlier than clinical judgment (15–17). However, due to its high sensitivity to the surrounding environment, small changes in room temperature may remarkably affect the infrared thermography signal from the wound (18). Laser doppler with low-resolution fast scan is reported to be more accurate and effective than clinical judgment alone in predicting burn wound outcome. The laser light in wound tissue exhibits a frequency change, which is correlated with the amount of tissue perfusion (19–21). It is worth noting that the high cost of the laser doppler imaging device hinders its wide clinical application. Besides this, any body movement during the scanning could result in inaccurate imaging (22).

ICG angiography, as a widely applied fluorescence imaging technique, can provide confirmatory information of tissue perfusion in various kinds of flap surgery and affect decision making about skin sparing to minimize the potential of necrosis. Driessen et al. (23) studied female patients undergoing mastectomies with or without immediate reconstruction and revealed a substantial decrease in skin necrosis with the use of ICG angiography. Kawamoto et al. (24) report that, compared with thermography, ICG angiography could confirm variation in perfusion of the intercostal muscle flap and make it possible for reducing bronchopleural fistula development. Abdelwahab et al. (25) notes that ICG angiography is an effective method to qualify and quantify neovascularization perfusion of forehead flaps, and time between stages and cartilage graft use were significantly associated with perfusion measures. However, the application of ICG on the prediction of extent and perfusion of traumatic wounds is still lacking.

To our knowledge, this study is the first clinical research to demonstrate a decreased risk of trauma-related necrosis after ICG angiography evaluation in children. Our analysis showed that 15 cases (78.9%) experienced inconsistent judgment on skin perfusion by both clinical findings and ICG angiography. All intraoperative decisions in skin sparing were based on ICG results, and no postoperative necrosis was observed in 18 cases (94.7%). This could provide surgeons with objective evidence, which was beneficial for judgment on perfusion, the surgical plan, and prediction of prognosis. Furthermore, postoperative imaging software was performed to quantify tissue perfusion as a percentage, which helped to objectively manifest the distribution of blood supply in the traumatic wound. By studying the comparison between clinical appearance and postoperative software results, it can facilitate a surgeon's ability to judge intraoperative skin sparing in the future. Due to the short plasma half-life time of ICG, intraoperative ICG angiography with a 20-min interval could be repeated if necessary, and no ICG-related side effect was observed in this study.

The weaknesses of this research includes its small sample size and lack of prospective control study. Due to incompliant physical examination of children, ICG angiography can only be performed under general anesthesia. In addition, perfusion of deep tissue cannot be assumed by ICG angiography (limited to 10 mm for visualization), and thus, postoperative hypoperfusion of deep fat tissue might be the cause of one case with partial necrosis. Besides this, given that randomness of acute trauma (e.g., location, cause, extent, type), the control group without ICG evaluation was not included in our research.

In conclusion, our findings support the likelihood that ICG angiography renders a promising adjunctive technique for surgeons to make reasonable decisions in skin sparing in pediatric acute trauma.

## Data Availability Statement

The original contributions presented in the study are included in the article/supplementary material, further inquiries can be directed to the corresponding author/s.

## Ethics Statement

The studies involving human participants were reviewed and approved by Ethics Committee of the Children's Hospital of Nanjing Medical University. Written informed consent to participate in this study was provided by the participants' legal guardian/next of kin. Written informed consent was obtained from the individual(s), and minor(s)' legal guardian/next of kin, for the publication of any potentially identifiable images or data included in this article.

## Author Contributions

WS revised the manuscript and approved the final manuscript as submitted. JC performed the surgery and conducted the data analyses. BS and WW performed postoperative follow-up and analyzed the data. TH wrote a draft of the article and edited the figures. All authors contributed to the article and approved the submitted version.

## Conflict of Interest

The authors declare that the research was conducted in the absence of any commercial or financial relationships that could be construed as a potential conflict of interest.

## Publisher's Note

All claims expressed in this article are solely those of the authors and do not necessarily represent those of their affiliated organizations, or those of the publisher, the editors and the reviewers. Any product that may be evaluated in this article, or claim that may be made by its manufacturer, is not guaranteed or endorsed by the publisher.
